# Chemical Composition, and Antioxidant and Antimicrobial Activities of Essential Oil of *Phyllostachys heterocycla* cv. *Pubescens* Varieties from China

**DOI:** 10.3390/molecules16054318

**Published:** 2011-05-24

**Authors:** Yong-Chun Jin, Ke Yuan, Jing Zhang

**Affiliations:** 1 The Nurturing Station for the State Key Laboratory of Subtropical Silviculture, Zhejiang Agriculture and Forestry University, Lin’an, 311300, China; 2 College of Pharmacy, Henan University of Traditional Chinese Medicine, Zhengzhou 450008, China

**Keywords:** *Phyllostachys heterocycla* cv*. Pubescens*, essential oil, GC/MS, anti-oxidant, anti-microbial

## Abstract

The essential oils of *Phyllostachys heterocycla* cv. *Pubescens*, *Phyllostachys heterocycla* cv. *Gracilis*, *Phyllostachys heterocycla* cv. *Heterocycla* and *Phyllostachys kwangsiensis* leaves were obtained by steam distillation. Their chemical components were separated and identified by gas chromatography/mass spectrometry (GC/MS). Meanwhile, the effect of scavenging free radicals of essential oil was assayed by using the DPPH·method with Trolox^®^ as control to evaluate their antioxidant capacities. Gram-positive (*Staphyloccocus aureus*) and Gram-negative (*Escherichia coli*) were selected as the indicator microorganisms to evaluate the antimicrobial activity. Antimicrobial properties were estimated by the agar diffusion method. The results show that 63 components were separated and identified by GC/MS from these varieties of bamboo leaves. *cis*-3-Hexenol, whose content in cv. *Pubescens*, *Gracilis*, *Heterocycla* and *Ph. kwangsiensis* was 27.11%, 24.62%, 30.51% and 34.65%, respectively, was the main constituent. The relative content of alcohol compounds in these varieties of essential oils ranged from 39.8% to 46.64%. All of the bamboo leaf essential oils possessed certain antioxidant capacity; the corresponding IC_50_ values were 3.1622, 4.9353, 4.2473, and 5.4746 μL/mL, respectively. Essential oils of all tested bamboo spp. were active against *Staphylococcus epidermidis* and *E. coli*, showing a positive correlation with the essential oil concentration of 50.42-300 μL/mL. The results indicated there were no significant differences among three varieties and the related species with respect to their antioxidant and antimicrobial activities. This paper provides evidence for studying the essential composition from different varieties of bamboo leaves.

## 1. Introduction

*Phyllostachys heterocycla* cv*. Pubescens* which accounts for about 70% of the bamboo in South China [1], is one of the most important economic bamboo resources, due of its many advantages such as rapid growth rate, high yield, extensive use, short crucial period for forest formation and strong regeneration capacity [2].

The essential oils of the genus, which are a secondary metabolism product, have been widely used in nonmedicinal applications such as a flavoring agent (condiment and spice) in food [3]. There is evidence on the antioxidant [4,5], free radical scavenging [6], antibacterial [7,8] and antiparasitic activities of the essential oils, but *Phyllostachys heterocycla* cv*. Pubescens* encompasses different varieties with variable chemical constituents due to inter-specific hybridization, and the number of *Phyllostachys heterocycla* cv*. Pubescens* varieties is reported to be more than 10 [9]. Because all these species look very similar and are difficult to distinguish, the antimicrobial and antioxidant activities of the essential oils of the different varieties have never been reported before. Consequently, the purpose of the present investigation was to analyze the chemical constitution, antimicrobial and antioxidant activities of the essential oils of plant material from different varieties of *Phyllostachys heterocycla* cv*. Pubescens*.

The essential oils of leaves from three cultivars of *Phyllostachys heterocycla* (cv. *Pubescens, Gracilis, Heterocycla*) and one plant related to this species (*Ph. kwangsiensis*) were obtained by steam distillation. Their chemical components were separated and identified by gas chromatography/mass spectrometry (GC/MS). Meanwhile, to evaluate the antioxidant capacities of the essential oils their free radical scavenging effect was assayed using the DPPH method with Trolox^®^ as control. To evaluate the antimicrobial activity *Staphyloccocus aureus* and *Escherichia coli* were selected as the indicator microorganisms. The results of this research constitute the first systematic analysis of chemical constituents in essential oils from bamboo leaves, providing a theoretical basis for the development and utilization of these plants.

## 2. Results and Discussion

### 2.1. Chemical Composition of the Essential Oils

**Figure 1:**
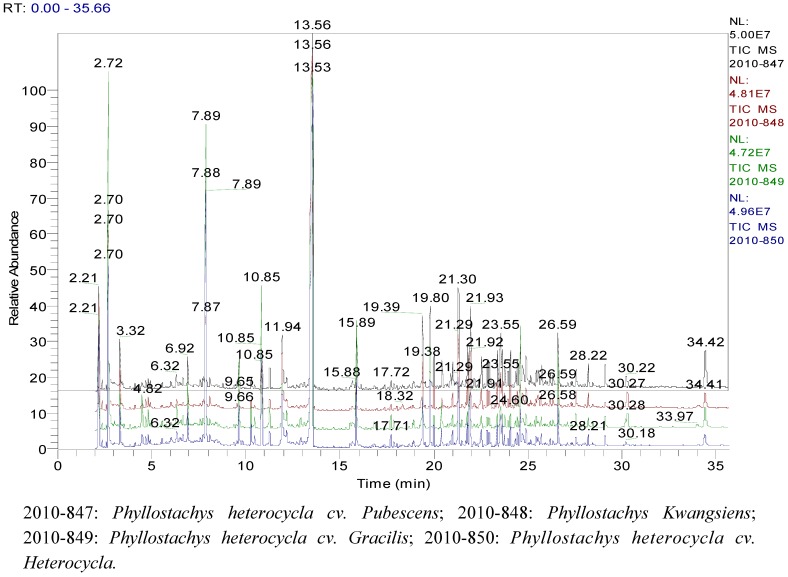
GC/MS total ion current chromatogram of the essential oils of the four samples.

The TIC of the essential oils of the four varieties was acquired (Figure 1) and analyzed to identify the components by MS. After scanning each peak, the mass spectrum was obtained and the relative contents of the constituents of the essential oils were determined by the area normalization method. 

Identification of individual compounds was made by comparison of the internal reference mass spectra library, with authentic compounds or with data reported in the literature. The identified compounds are listed in Table 1.

**Table 1:** Chemical composition of the essential oils of the four samples.

No.	Name of components	M.F.	RT	A %	B %	C %	D %
1	2-Hydroxy-2-methylpropanenitrile	C_4_H_7_NO	2.17	--	--	0.83	--
2	2-Methoxyethyl acetate	C_5_H_10_O_3_	2.17	--	--	--	1.14
3	2-Methyl-2-butenal	C_5_H_8_O	2.21	2.15	--	2.46	3.08
4	Butyraldehyde	C_4_H_8_O	2.21	--	3.14	--	--
5	Butyric acid	C_4_H_8_O_2_	2.70	0.22	0.24	0.47	--
6	3-Methyl-2-butanol	C_5_H_12_O	2.98	6.80	7.81	13.76	6.21
7	4-Hydroxy-2-butanone	C_4_H_8_O_2_	3.32	1.39	1.47	0.91	1.42
8	Octanoic acid	C_8_H_16_O_2_	4.48	--	0.36	1.13	0.66
9	Toluene	C_7_H_8_	4.82	0.28	0.40	0.49	0.37
10	Nonanoic acid	C_9_H_18_O_2_	6.32	0.46	0.37	0.85	0.55
11	1-Penten-3-ol	C_5_H_10_O	6.92	0.84	0.56	1.25	1.10
12	1-Hexanol	C_6_H_10_O	7.59	0.21	--	0.26	0.31
13	(*E*)-2-Hexenal	C_6_H_10_O	7.88	5.32	3.01	8.59	7.67
14	Capric acid	C_10_H20_2_	8.10	0.26	0.42	0.25	0.41
15	2-Penten-1-ol	C_5_H_10_O_2_	9.66	0.95	0.66	1.90	1.25
16	Undecanoic acid	C_11_H_22_O_2_	10.31	0.43	0.40	0.78	0.92
17	Dodecanoic acid	C_12_H_24_O_2_	10.85	1.37	1.88	4.96	3.39
18	2-Hexen-1-ol	C_6_H_12_O	11.25	--	--	0.76	--
19	Cyclohexanol	C_6_H_12_O	11.27	0.77	0.49		0.64
20	Salicylic acid methyl ester	C_8_H_8_O_3_	11.94	2.37	3.48	2.28	3.31
21	Furfural	C_5_H_4_O_2_	12.19	0.41	0.52	0.68	0.91
22	*cis*-3-Hexenol	C_6_H_12_O	13.55	27.1	34.65	24.62	30.51
23	Ethylbenzene	C_8_H_10_	15.68	--	--	0.27	0.45
24	Tridecanoic acid	C_13_H_26_O_2_	15.89	1.64	1.08	3.00	2.27
25	5-Ethyl-2(5H)-furanone	C_6_H_8_O_2_	17.72	0.28	0.21	0.42	0.42
26	Vanillyl alcohol	C_8_H_10_O_3_	18.32	--	0.40	--	--
27	2,5,5-Trimethyl-3-phenylcyclohexanone	C_15_H_20_O	19.39	2.42	2.18	1.17	1.90
28	Cedrol	C_14_H_28_O_2_	19.80	2.55	1.66	2.45	2.31
29	2-Phenylacetic acid	C_8_H_8_O_2_	20.00	1.07	0.91	0.45	0.75
30	Phytol	C_15_H_26_O	20.43	0.79	0.41	0.75	0.59
31	Tetradecoic acid	C_14_H_28_O_2_	20.88	0.54	--	--	0.25
32	Nonaldehyde	C_9_H_18_O	21.01	0.79	0.64	0.38	0.52
33	Hexadecanoic acid	C_16_H_32_O_2_	21.30	3.19	2.93	1.60	2.52
34	2-Ethylhexyl acetate	C_10_H_20_O_2_	21.49	0.32	0.26	0.32	0.22
35	Vamillic aldehyde	C_8_H_8_O_3_	21.79	1.28	1.10	0.52	0.87
36	1H-indole	C_8_H_7_N	21.92	3.19	2.76	1.43	2.22
37	1,7-Dimethyl-naphthalene	C_12_H_12_	22.15	0.43	0.32	--	0.29
38	1,6-Dimethyl-naphthalene	C_12_H_12_	22.52	1.32	1.10	0.51	0.84
39	2-(1-Methylethyl)-naphthalene	C_13_H_14_	22.82	0.82	0.75	0.32	0.55
40	1,4-Dimethyl-naphthalene	C_12_H_12_	22.95	0.86	0.79	0.41	0.53
41	4-Methyl-1,1'-biphenyl	C_13_H_12_	23.37	0.99	0.87	0.40	0.66
42	2,2'-Dimethylbiphenyl	C_14_H_14_	23.55	--	--	--	1.51
43	6,6-Dimethyl-2-methylene-bicyclo[3.1.1] heptane	C_10_H_16_	23.55	2.25	1.97	0.95	--
44	1-Ethyl-naphthalene	C_12_H_12_	23.62	1.17	0.94	0.50	0.79
45	2,6,6-Trimethylcyclohexa-1,3-dienecarbaldehyde	C_10_H_14_O	23.77	0.52	0.45	--	0.33
46	2,6,6-Trimethylcyclohex-1-enecarbaldehyde	C_10_H_16_O	23.93	0.51	0.34	0.24	0.31
47	1,4,6-Trimethyl-naphthalene	C_13_H_14_	24.07	1.10	0.98	0.43	0.70
48	1,4,5-Trimethyl-naphthalene	C_13_H_14_	24.35	0.54	0.44	0.23	0.33
49	4-Hydroxy-3-methoxy-styrene	C_9_H_10_O_2_	24.44	0.78	0.61	0.31	--
50	*α*-Ionone	C_13_H_20_O	24.61	1.14	0.85	2.36	1.04
51	1,1'-Methylenebis-4-methylbenzene	C_15_H_16_	24.70	0.72	0.59	0.30	0.49
52	4,4'-Dimethylbiphenyl	C_14_H_14_	24.76	0.59	0.52	0.27	0.39
53	Trimethylcyclohexadienylbutenone	C_13_H_18_O	24.89	0.98	0.96	--	0.73
54	1-Methyl-3-(phenylmethyl)benzene	C_14_H_14_	25.54	0.77	0.71	0.21	0.29
55	(*E*)-1,2,3-Trimethyl-4-(prop-1-en-1-yl) naphthalene	C_16_H_18_	25.73	0.54	0.41	0.26	0.36
56	Hexadecanoic acid	C_16_H_24_O_2_	26.59	1.33	0.88	1.70	1.08
57	6,10,14--Trimethyl-2-pentadecane	C_18_H_10_O	27.55	0.56	0.21	0.37	0.27
58	9-Octadecenal	C_18_H_34_O	27.28	--	0.59	--	--
59	1,2-Benzenedicarboxylicacid-bis(2-methylpropyl) ester	C_16_H_22_O_4_	28.22	0.69	0.54	0.31	0.40
60	*β*-Ionone	C_13_H_20_O	29.11	0.87	0.30	0.42	0.45
61	Tetradecanoic acid	C_14_H_28_O_2_	30.22	0.63	--	0.34	--
62	Dibutyl phthalate	C_24_H_38_O_4_	30.28	--	1.00	0.58	--
63	Octacosane	C_28_H_58_	34.42	2.68	1.42	1.36	1.07
	Total identified			92.19	92.94	92.77	92.55

A: *Phyllostachys heterocycla cv*. *Pubescens*; B: *Phyllostachys kwangsiensis*; C: *Phyllostachys heterocycla cv*. *Gracilis*; D: *Phyllostachys heterocycla cv. Heterocycla.*

In all 63 constituents were identified in the essential oils (Table 1) with 53 (representing 92.19% of the total amount) being found in cv. *Pubescens*; 53 (representing 92.77% of the total amount) in cv. *Gracilis*; 53 (representing 92.55% of the total amount) in cv. *Heterocycla*, and 54 (representing 92.94% of the total amount) in *Ph. kwangsiensis*. *cis*-3-Hexenol and 3-methyl-2-butanol were the main constituents; this has also been reported elsewhere [3]. The contents of *cis*-3-hexenol for cv. *Pubescens*, *Gracilis*, *Heterocycla* and *Ph. kwangsiensis* were 27.11%, 24.62%, 30.51% and 34.65%, respectively, and the contents of 3-methyl-2-butanol were 6.8%, 13.76%, 6.21% and 7.81%, respectively. For (*E*)-2-hexenal the values were 5.32% (cv. *Pubescens*), 8.59% (cv. *Gracilis*) and 7.67% (cv. *Heterocycla*). The other major compounds were *n*-hexadecanoic acid (3.19%), 1*H*-indole (3.19%) in cv. *Pubescens* and dodecanoic acid (4.96%) for cv. *Gracilis*. However, the trend was somehow different for *Ph. kwangsiensis*, where the content of salicylic acid methyl ester was 3.48%. 

### 2.2. Antioxidant Activity

The DPPH assay has been widely used to test the free radical scavenging ability of plants. The essential oils of four varieties and the synthetic antioxidant Trolox^®^ were set up six different concentrations to determine the antioxidant activity with DPPH method. The result was analyzed by regression analysis. The DPPH scavenging activities (IC_50_) of the essential oil of four varieties and the regression equation are presented in Table 2.

**Table 2:** Antioxidant activity of the essential oils of the four samples*.*

Samples	Regression equation	R^2^	IC_50 _(µL/mL)
Phyllostachys heterocycla cv. Pubescens	y = 0.0339x + 0.3928	0.9979	3.1622
Phyllostachys heterocycla cv. Gracilis	y = 0.0201x + 0.4008	0.9579	4.9353
Phyllostachys kwangsiensis	y = 0.0236x + 0.3708	0.9797	5.4746
Phyllostachys heterocycla cv. Heterocycla	y = 0.0283x + 0.3798	0.9793	4.2473
Trolox^®^	y = 7..8087x + 0.2975	0.9935	0.0259 mg/mL

According to this study, the concentration ranges in which the oils display effective antioxidant activity are not the same, but the scavenging effects increased with the increase of sample concentration within their proper concentration range. In addition, the free radicals will be completely scavenged if the concentration is too high, and scavenging activities cannot be detected very well if too low, therefore, for comparing the antioxidant activity of these substances, the IC_50_ value, defined as the concentration of the antioxidant required to scavenge 50% of DPPH free radical, was used as indicator of free radical scavenging ability. The IC_50_ is a parameter often used to evaluate antioxidant activity. In our study, the Trolox^®^ solutions were used for calibration, and the standard curve was obtained using Trolox^®^ concentration (0.0102~0.0612 mg/mL) as the abscissa axis and absorption values as the vertical axis, y = 7.8087x + 0.2975, (r^2^ = 0.9935). There was a good linear relationship between the free radical scavenging rate and the concentration of oil in the ranges 0.30~10.0 μL/mL. The results indicated that there was a significant positive correlation between the free radical scavenging rate and the essential oil concentration. The essential oils of the four varieties all have the DPPH free radical scavenging activity that in terms of IC_50_ values can be ranked as follows: *Ph. kwangsiensis*> cv. *Gracilis*> cv. *Heterocycla*> cv. *Pubescens*. In a word, cv. *Pubescens* could be a promising source of natural antioxidants. As for which composition has more significant impact, further studies on the antioxidant mechanism are needed in the future.

### 2.3. Antimicrobial Activity

Gram-positive (*Staphyloccocus aureus*) and a Gram-negative (*Escherichia coli*) were selected to evaluate the antimicrobial activity of the essential oils. The antimicrobial properties were estimated by the agar diffusion method [12]. The maximum inhibition zones [13] and MIC values [14] of the bacteria are shown in Table 3 and Table 4. The results indicated that the essential oils of all tested plants were active against *Staphylococcus aureus* and *E. coli*, showing a positive correlation to the essential oils’ concentration.

**Table 3:** Antimicrobial activity of the essential oils of the four samples given as inhibition zone (measured in mm)^a^.

Organism	Dose, µL/disk	Samples of essential oil (IZ, mm)			
		A	B	C	D
*Escherichia coli*	300	26.09	29.37	28.77	21.93
	210	20.34	26.49	26.99	19.86
	147	15.68	21.21	21.73	18.56
	102.9	13.84	20.37	15.38	9.04
	72.03	6.79	18.00	10.45	7.14
	50.42	6.10	13.29	9.68	6.18
*Staphyloccocus aureus*	300	20.15	31.02	30.34	10.85
	210	19.31	27.65	27.19	9.05
	147	12.43	23.54	26.59	7.18
	102.9	10.79	21.33	19.79	6.13
	72.03	7.61	20.43	15.67	6.06
	50.42	8.58	18.20	10.30	5.17

^a^ Mean value, n = 3.

**Table 4:** Antimicrobial activity of the essential oils of the four samples given as minimum inhibitory concentration.

Organism	Samples of essential oil (MIC, µL/mL)			
	A	B	C	D
*Escherichia coli*	35.29	35.29	35.29	35.29
*Staphyloccocus aureus*	35.29	24.70	24.70	50.27

The maximum inhibition zones and MIC values showed that *E. coli* and *Staphylococcus aureus* were sensitive to the essential oils of the four varieties*.* The *Ph. kwangsiensis* and cv. *Gracilis* oils had the same effect against *Staphylococcus aureus* and *E. coli*, but the activity of cv. *Pubescens* and cv. *Heterocycla* was different, as *E. coli* was much more sensitive towards these oils than *S. aureus*. The sequence of antimicrobial activity was: *Ph. Kwangsiensis* > cv. *Gracilis* > cv. *Pubescens* > cv. *Heterocycla.* The MIC values showed significantly different activities among the different varieties. These differences among antimicrobial activities of these four essential oils could be due to different amounts of alcohols present in each oil.

## 3. Experimental

### 3.1. Materials

The fresh leaves of *Phyllostachys heterocycla* cv. *Pubescens*, cv. *Gracilis*, cv. *Heterocycla* and *Ph. kwangsiensis* were collected at the Anji Bamboo Garden of Zhejiang Province, China. The species were identified by Xin-chun Lin from Zhejiang Agricultural and Forestry University. 2,2-Diphenyl-1-picrylhydrazyl (DPPH) and 6-hydroxy-2,5,7,8-tetramethychroman-2-carboxylic Acid (Trolox^®^) were obtained from the Beijing Qi-hua Bioengineering Institute (Beijing, China). *Escherichia coli* and *Staphylococcus aureus* were provided by the Microorganism Laboratory of Zhejiang Agriculture and Forestry University.

### 3.2. Isolation of Essential Oil

Air-dried and finely crushed (to pass through a no. 40 sieve) leaves of the plant (100 g) were extracted successively with water (1000 mL) by steam distillation according to the essential oil procedure of the *2005 Chinese Pharmacopoeia* [10], giving a yellow oil which was dried with anhydrous sodium sulfate and stored in a sterilized vial at 4 °C until analysis by gas chromatography-mass spectrometry (GC/MS). Yield percentage was calculated as volume (mL) of essential oil per 100 g of plant dry matter. The results showed that the essential oil contents in different varieties ranged from 1.3% to 1.8%, with the highest being obtained from *Ph. kwangsiensis* (1.8%) and the lowest in cv. *Gracilis* (1.3%). The essential oil contents of cv. *Pubescens* and cv. *Heterocycla* were 1.5%, and 1.6%, respectively.

### 3.3. Gas Chromatography/Mass Spectrometry (GC/MS) Analysis

GC/MS analysis was carried out using splitless injection mode on a Varian CP3800/1200L GC-MS instrument, equipped with a HP-5 fused silica capillary column (phenylmethylsiloxane, 30 m × 0.25 mm, 0.25 μm film thickness). Helium was used as the carrier gas, at a flow rate of 0.8 mL/min. Oven temperature was programmed at 45 °C for 3 min, then 45-90 °C at 10 °C/min, then 90-180 °C at 6 °C/min, then 180-230 °C at 12 °C/min, then 230-250 °C at 9 °C/min and finally held at 250 °C for 9 min. The injector and detector temperature were set at 250 °C and 280 °C, respectively. The electron impact source was 70 eV, ion source temperature was 200 °C, the mass range 33-450 amu and the scan rate was 0.5 s. The components of the essential oils were identified by comparing their mass spectral fragmentation patterns with those of similar compounds from databases (NIST and Wiley Mass Spectral Libraries). For each compound on the gas chromatogram, the percentage of peak area relative to the total peak area of all compounds was determined and reported as relative amount of that compound, without using correction factors. 

### 3.4. Antioxidant Activity AssayInfinite M 200 Universal Microplate Spectrophotometer (Swiss Tecan company, Swiss)

The free radical scavenging activity of the extract was measured in terms of hydrogen donating or radical scavenging ability using the stable free radical DPPH [2]. An aliquot (100 µL) of each sample (with different concentrations) was added to DPPH solution (41.6 mg/L, 200 μL) in ethanol. Discoloration was measured at 517 nm using after the contents were mixed and allowed to stand at 24 °C for 30 min (Infinite M 200 Universal Microplate Spectrophotometer (Swiss Tecan company, Swiss). IC_50_ value (the concentration required to scavenge 50% DPPH free radicals) was calculated. Trolox^®^ was used as the positive control. The capability to scavenge the DPPH radical was calculated using the following equation:

DPPH Scavenging effect (%) = 1 − (A_sample_ − A_blank_) / A_control_ × 100%

where ethanol (200 μL) plus sample solution (100 μL) was used as a blank and DPPH-ethanol solution (200 μL) plus ethanol (100 μL) was used as a negative control [11,15].

### 3.5. Antimicrobial Activity Assay

Antimicrobial properties were estimated by the agar diffusion method using filter paper disks. Bacteriostatic annulus diameters were used to judge bacteriostatic ability [12]. The essential oil was serially diluted to 50.42, 72.03, 102.9, 147, 210, and 300 µL mL^-1^ in ether. The microorganism suspensions were finally diluted to 10^7^~10^8^ Colony-Forming Units (CFU) mL^-1 ^for use in the assays. Plates containing ether in medium served as solvent controls. The plates were incubated at 30 °C for 24 h before the measurement of inhibition zones diameters. Minimal inhibitory concentration (MIC) was determined as the lowest concentration of the essential oils able to completely inhibit visible growth of the microorganism, as detected by the naked eye [16,17]. Each test was performed in triplicate. 

## 4. Conclusions

This paper describes a comparative study of chemical composition, antioxidant and antimicrobial activity of essential oil obtained from the leaves of four varieties of *Phyllostachys pubescens* from China. The three varieties and one related species of *Phyllostachys pubescens* are very similar in shape and influenced by environment in their process of growth, which may cause chemical changes. In light of the results obtained, we can conclude that there is obvious difference in the relative contents of chemical constituents among the four varieties’ essential oils, although their main constituents were all similar and differed slightly in composition. This study used the DPPH method to evaluate their antioxidant capacities. The result indicated that all these essential oil possessed antioxidant activity, and it there was not obvious difference between the activities of the essential oils of the four varieties. The sequence of antioxidant activities from best to the worst was cv. *Pubescens* > cv. *Heterocycla* > cv. *Gracilis* > *Ph. kwangsiensis.* This paper studied antimicrobial activity of essential oils of four varieties against Gram-positive and Gram-negative bacteria, and according to the results, all of these essential oils possessed antimicrobial activity. The degree of antimicrobial activity could be ranked as follows: *Ph. Kwangsiensis* > cv. *Gracilis* > cv. *Pubescens* > cv. *Heterocycla.* The results also indicated that there were no significantly differences among antioxidant and antimicrobial activities of the two species.

There is a trend to find antioxidant and antibacterial materials from natural products in the modern medical industry. The above results show that the essential oils of bamboo leaves could be a potential source of compounds with antioxidant and antibacterial activities and the results provide a reference point for further research on the chemical components of bamboo leaf volatile oils as well as for their utilization.
